# Visual Leakage Inspection in Chemical Process Plants Using Thermographic Videos and Motion Pattern Detection

**DOI:** 10.3390/s20226659

**Published:** 2020-11-20

**Authors:** Mina Fahimipirehgalin, Birgit Vogel-Heuser, Emanuel Trunzer, Matthias Odenweller

**Affiliations:** 1 Institute of Automation and Information Systems, Technical University of Munich, 85748 Garching, München, Germany; vogel-heuser@tum.de (B.V.-H.); emanuel.trunzer@tum.de (E.T.); 2 Evonik Technology and Infrastructure GmbH, 63457 Hanau, Germany; matthias.odenweller@evonik.com

**Keywords:** leakage detection and localization, anomaly detection, image analysis, Kalman filter, motion pattern detection, multi-object tracking

## Abstract

Liquid leakage from pipelines is a critical issue in large-scale chemical process plants since it can affect the normal operation of the plant and pose unsafe and hazardous situations. Therefore, leakage detection in the early stages can prevent serious damage. Developing a vision-based inspection system by means of IR imaging can be a promising approach for accurate leakage detection. IR cameras can capture the effect of leaking drops if they have higher (or lower) temperature than their surroundings. Since the leaking drops can be observed in an IR video as a repetitive phenomenon with specific patterns, motion pattern detection methods can be utilized for leakage detection. In this paper, an approach based on the Kalman filter is proposed to track the motion of leaking drops and differentiate them from noise. The motion patterns are learned from the training data and applied to the test data to evaluate the accuracy of the method. For this purpose, a laboratory demonstrator plant is assembled to simulate the leakages from pipelines, and to generate training and test videos. The results show that the proposed method can detect the leaking drops by tracking them based on obtained motion patterns. Furthermore, the possibilities and conditions for applying the proposed method in a real industrial chemical plant are discussed at the end.

## 1. Leakage Inspection in Chemical Process Plant

Pipe networks are one of the most important elements of chemical process plants and a reliable condition monitoring of pipelines is indispensable for safe transportation of toxic or hazardous chemical substances. Considering the high maintenance cost [1] as well as safety demands [2], the ability to detect and localize failure in pipelines at an early stage has become a critical issue. Based on a study [2], the risk level of hazardous situations caused by leakage of poisoning substances from the pipelines is unacceptable.

The conventional leakage inspection in chemical process plants relies on human operators, which is highly dependent on the competency of the operator and the frequency of the inspection. Therefore, an automatic leakage inspection mechanism is required to permanently monitor the plant, to detect and localize the leaking drops [3]. One promising approach for automatic leakage monitoring is using thermal infrared (IR) cameras [4]. There are several applications of these cameras in real industrial environments in recent years [5]. They have several advantages, e.g., high accuracy and high speed in recording the images.

In the case of leakage detection, IR cameras can capture the effect of leaking drops independent of the physical properties of the substances inside the pipelines, pipeline materials, shapes, and sizes [4]. Furthermore, the use of IR cameras can be very practical in capturing the effect of comparatively small leaking drops, if the temperature difference between the liquid and the environment is high enough. IR cameras usually can capture a minimum temperature difference in the range of 273.2−273.25 K [4], and, therefore, if the temperature difference of leaking drops satisfies this minimum range, they can be observable in thermographic images. Most chemical processes, e.g., crude oil refining, work with liquid streams with higher temperature than the environment [6]. Therefore, the required temperature difference for leakage detection using IR cameras is often satisfied in this domain and, thus, they are applicable for leakage detection. Using IR cameras and taking advantage of machine vision techniques [7,8] provide an automatic vision-based system for leakage monitoring in large-scale chemical process plants.

The main contribution of this paper is to propose a method for liquid leakage detection in chemical process plants Using IR cameras. The leaking liquid drops are considered as moving objects in the IR video data, and leakage detection and localization are done by a multi-object tracking approach in the video data. The motion pattern of a single drop is tracked using the Kalman filter in training videos and the leaking drops are differentiated from noise if their motion patterns can be predicted by learned Kalman filters from training data.

The rest of the paper is structured as follows: In Section 2, the requirements for an efficient leakage detection system are discussed. Section 3 provides an overview of recent studies in leakage detection and investigates them regarding the defined requirements. Furthermore, in this section, an overview of recent approaches in object tracking and motion pattern detection in image and video data is provided as well. Section 4 describes the detailed steps of the proposed method using IR video data of a laboratory demonstrator plant. The evaluation of the proposed method and the results are given in Section 5. In order to evaluate and compare the performance of the proposed approach, the particle filter as a non-parametric and baseline method is also applied for leakage tracking. Finally, the conclusion and outlook are discussed in Section 6.

## 2. Requirements for Efficient Leakage Detection

In order to provide an efficient and applicable leakage detection mechanism to identify leakages in the early stage before leading to a hazardous situation, some requirements should be met. To guarantee the safety aspects in an industrial environment and have a reliable leakage detection mechanism, the proposed method should have a high accuracy (Requirement R1) and be robust against the noise in the environment (Requirement R2).

**Figure 1:**
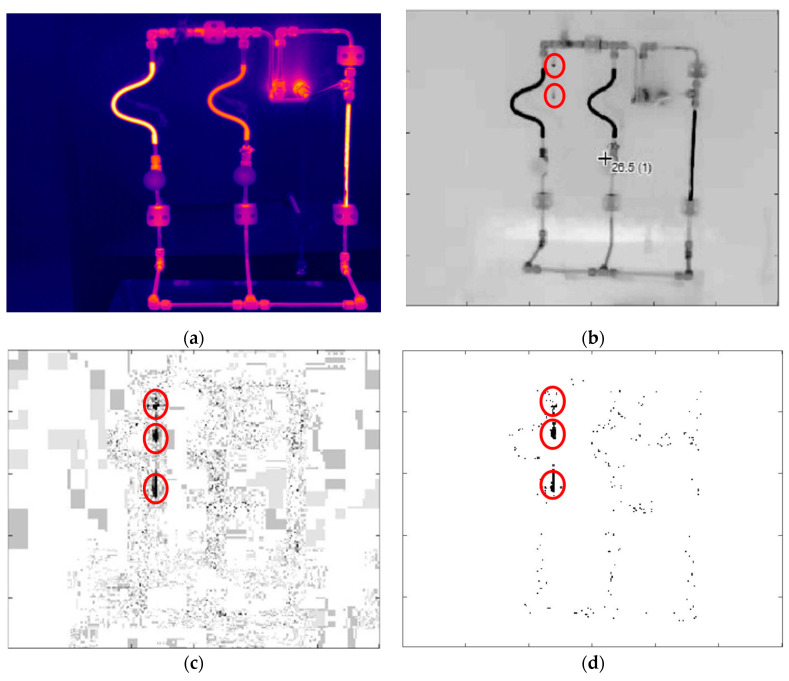
(**a**) Raw infrared image of the demonstrator plant used in this experience. (**b**) Sample frame of the demonstrator plant with leakage resulting from the MP4 format in gray scale. (**c**) Subtracted frame resulting from subtracting subsequent frames. (**d**) Subtracted frame after background noise removal. After background noise removal, the noise along the pipes still remains. Leaking drops are marked with solid line circles.

In noisy environments, the detection of small drops and the differentiation from the noise is a crucial task since the small leaks can be neglected or removed as noise in the video. Therefore, the proposed method should be able to detect small drops and differentiate them from noise as well (Requirement R3). In recent existing studies, there is a focus on small leakage detection. In comparison with a large leakage area, small leakages do not make significant changes in the pressure and flow of the liquid and, therefore, it is difficult to detect them [9,10,11]. In liquid leakage detection, a clear definition of small drops is challenging and existing literature measures the size of leaking drops differently. In general, small leaks usually refer to a situation that pressure changes related to leakage are relatively small concerning the levels of noise and the whole pressure range [9]. However, in the recent leakage study, different metrics are defined to measure the size of the leakage. Ostapkowicz defines the small leakage as a certain percentage of the whole flow rate in the pipes [9], Liu et al. define the small leakage as a certain ratio of leakage orifice to pipe diameter [11], He et al. define a certain leakage volume and amount of liquid that exits from the orifice as a measure for small leakages [10]. In this paper, the size of the leakage is considered in the number of pixels in the images in the vision-based inspection method. A small leak is considered as the minimum number of pixels that form a leaking drop in the image which make a single leaking drop observable in the infrared image. Furthermore, in this paper a demonstrator plant is used as a testbed, in which the entire plant is observable in each image (see Figure 1a).

As another important requirement for leakage detection, the detection of multiple simultaneous leakages should be addressed as well (Requirement R4). In large-scale chemical process plants, there are cases that multiple leakages from different positions of the plant occur simultaneously. Detection of multiple simultaneous leakages based on existing methods requires sophisticated sensor equipment [12] and there is still a research gap in efficient and practical leakage detection in the case of multiple and simultaneous leakages.

Beside the leakage detection, leakage localization (Requirement R5) is another important issue in leakage monitoring to direct to the right place of the leakage and avoid further disturbances in that area. Finally, in order to be able to extend the method to other types of liquids and pipeline materials and shapes, the leakage inspection method should be independent of the physical properties of the liquid in the pipes and physical characteristics of the pipelines (Requirement R6). In the following section, the existing methods in leakage detection are reviewed based on the defined requirements. The summary of the requirements is provided in Table 1.

## 3. State of the Art

In this section, the existing literature in leakage monitoring is first reviewed. Then, the existing approaches for motion pattern detection in image and video data are summarized.

### 3.1. State of the Art in Leakage Monitoring

Leakage detection methods have been extensively studied in recent years and applied in many industrial fields, such as water distribution, gas transportation, and chemical process plants. These methods range from manual inspection to sophisticated sensor equipment [12]. In some existing methods, a mathematical model based on the process parameters is derived to model the flow inside the pipeline. In these models, leakage can be detected when the physical behavior of substances inside the pipes deviates from the model. Model-driven methods are usually based on Negative Pressure Wave (NPW) [13], or based on analysis of amplitude attenuation of the pressure signal [11] to detect leakage. Modeling the equilibrium point for flow and pressure and detecting the leakage by the deviation of the model from the equilibrium point [14] is another approach for leakage detection. However, in these methods, profound knowledge of the process is required which is difficult to obtain in most of the cases [15], and in the case of multiple simultaneous leakages (R4), the mathematical model is very difficult to obtain. Furthermore, the accuracy (R1) of the methods based on NPW is highly dependent on the precision of the sensors [12]. Using Acoustic Emission (AE) sensors is another approach to model the process parameters and to detect the leakage [16,17]. However, this method is highly dependent on the material and the characteristics of the pipelines (R6) [12] and the accuracy of the method decreases when the leaking drops are very small (R1, R3) [4].

In some other existing approaches, mathematical models of process parameters are jointly used with process data to detect leakages. Optimization of the error between the measured pressure and computed pressure by the model [18], or signal processing of the measured pressure and comparison with the computed pressure [9,19] are common approaches in this case. However, these methods are not accurate enough (R1) to capture all dynamics of the flow in the pipelines [11]. Some other existing approaches in leakage detection are only based on data-driven methods using sensor data: for instance, measuring the vibration of the pipes using fiber optic sensors and classifying it based on normal and abnormal vibration signals [20]. However, fiber optic sensors are very sensitive to environmental noise (R2) [12]. Using a fuzzy classifier [21,22] to classify the residuals between measured flow and predicted flow is another approach; however, it is not applicable for small drops (R3) due to very small residuals between measured flow and predicted flow in this case.

Among exiting approaches for leakage detection, IR cameras are used in the literature to detect leakage in different fields, such as water distribution, gas transportation, and other process plants [4]. Nellis [23] used IR cameras for the first time to monitor water canals. Another application of IR cameras for leakage detection can be found in the work of Adefila et al. [24]. They evaluate the sensitivity of IR cameras in capturing temperature changes in the leaking area, but they do not apply further image processing for automatic leakage detection. Kroll et al. [25] use two-dimensional Gaussian distribution based on the temperature profile of a typical leak to model the leakage area in thermographic images. A similar approach is proposed by Dai et al. [26] to detect the region of leaking gas in IR images by the means of an improved Surendra algorithm. The methods introduced in [25,26] are also not suitable for the detection and localization of small leaking drops (R3), since they are more applicable for region-growing leaks such as the region of spreading liquid on a surface. Leaking drops from pipelines can be observed as an object moving along the pipes, and therefore have a different pattern than a region-growing leak pattern. Atef et al. [27] use IR images for an automatic leakage detection mechanism in water transportation pipes. For leakage localization, they propose a segmentation method based on the region-growing method, and the region with high temperature changes is marked as the area of the leakage. They consider the water leakage as spreading water on the surface which can be detected by region-growing pattern detection. An oil leakage monitoring system based on IR cameras and applied wavelet transformation on the images is proposed by Kuzmanic et al. [28] to detect leaking oil drops. However, their method is very sensitive to noise, and in the case of small drops of oil, it is difficult to distinguish them from noise (R2). Fahimipirehgalin et al. [29] propose a method based on PCA (Principle Component Analysis) and KNN (K-Nearest Neighbor) classification to classify the normal (no leakage) and abnormal (leakage) operations of a chemical plant using thermographic images. Their method can only detect the leakage but it cannot localize the leaking drops (R5). Furthermore, if the intensity of the noisy pixels is high, the proposed method will detect the noisy video as an abnormal (leakage) case. The noisy pixels with high intensity will affect the variance of the pixel values. Since the PCA method is highly dependent on the variance of the pixels’ value, in this method, noisy pixels with high intensity are detected as leakages.

### 3.2. State of the Art in Motion Pattern Detection

As mentioned before, in this paper, a method for leakage detection by identifying the motion patterns of the leaking drops in IR videos is proposed. In recent years, there are several studies in the analysis and modeling of motion patterns in image and video data. In this domain, different methods such as Kalman filter, particle filter, region-based, and active contour-based strategies are studied widely to track objects [30]. An adaptive motion tracking is introduced by Grimson et al., 1998 [31] in which the location, velocity, and size of the object are used as features to track the object based on the numeric iterative hierarchical clustering method. This method is not applicable for object tracking in the case of unknown size, location, and velocity of an object. Hu et al. [32] propose a system for learning motion patterns based on the hierarchical clustering of the spatial and temporal position of an object. However, the method has high computational complexity. Basharat et al. [33] represent the spatial state of the visual object over time using a Gaussian Mixture Model (GMM) and non-parametric representations of track statistics and use the detected motion patterns for abnormal motion detection. Since they consider some of the changes in the environment as part of the background, some motion patterns might be missing in their method.

One of the common approaches for object tracking in the literature is the particle filter [34]. This method is based on a set of samples using a sequential Monte Carlo method to represent and predict the probability density function of the system state and update it based on the latest measurement. This approach has high computational complexity and is more suitable for tracking nonlinear, non-Gaussian, and non-parametric motions [34]. The method requires several Monto Carlo simulations for the estimation of the next possible position of the tracked objects. Therefore, it is especially useful when the motion of the tracked object follows a chaotic model and several simulations are required to detect the next position. However, a particle filter is highly sensitive to measurement noise and requires quite a large number of initial particles to achieve a good tracking model [35].

If the state of the tracked object can be estimated by a linear model, the Kalman filter provides the optimum solution [34] and avoids additional optimization problems. Furthermore, due to the computational efficiency and robustness of the Kalman filter, it has been known as an efficient method for motion pattern detection [36] and multiple object tracking [37]. There are several studies in the literature which use a Kalman filter to track one or several objects in image or video data. Li et al. [38] use a Kalman filter for multi-object tracking for the human body and vehicle tracking. They consider a new Kalman filter motion tracking per each individual object and track them in a specific assigning tracking window. A similar approach for vehicle tracking is proposed by Shantaiya et al. [39], in which they improve the method for occlusion handling as well. In the methods proposed by Li et al. and Shantaiya et al. [38,39], object detection is based on the center of mass of a moving object. Chavan and R. Gengaje [40] propose an object detection method based on GMM and track the detected objects using a Kalman filter. However, if the number of objects is unknown, it is difficult to adapt GMM for object detection. An object tracking method based on the Kalman filter and the Hungarian data association algorithm is proposed in [41] for the crop detection system. In their method, first, the position of the new crop is detected by the Kalman filter, then the Hungarian algorithm is used to assign each detected position to a correct trajectory. However, in highly noisy environments with a lot of false alarms, the performance of the Hungarian data association will significantly deteriorate [42].

In this paper, to detect leaking drops from the pipelines in an IR video, they are tracked as multiple objects in the video. It is assumed that the motion of leaking drops from the pipelines can be estimated as linear motion in the vertical direction. Based on the reviewed literature, the Kalman filter is the most efficient method for tracking linear motions and, therefore, it can be a suitable method to track the leaking drops as multiple objects and localize them in the video data. Furthermore, leakage should be detected as fast as possible with low time complexity. As the Kalman filter has a low time complexity for tracking linear motions [36], it can provide fast leakage detection as well.

## 4. Leakage Detection Using Kalman Filter

In this section, the steps of leakage detection based on motion pattern detection using the Kalman filter method are discussed in detail. In order to evaluate the reliability of this method, a laboratory demonstrator plant along with an IR camera serve as a testbed.

### 4.1. Data Acquisition and Pre-Processing

**Figure 2:**
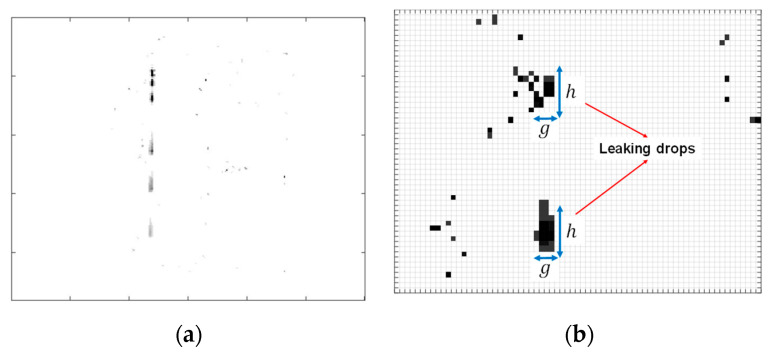
Object definition for leakage detection. (**a**) The fading problem in leakage detection. Leaking drops lose temperature, and therefore the intensity of the pixels gets less when they reach the bottom of the frame. (**b**) Zoomed area in a frame and object definition for leaking drops as a set of points with at least h and g non-zero neighbours in vertical and horizontal directions, respectively.

In the provided testbed, a laboratory demonstrator plant is assembled which includes a thermostat with an integrated pump to circulate water through a series of pipes. Leakage can be generated by losing some of the connectors in the pipes. An IR camera is used to capture video data of the demonstrator plant in normal (without leakage) and anomalous operations (including leakages). Among anomalous videos, the speed and positions of leakages are varied. All videos have a length of one minute and the frame rate is 25 frames per second, while the resolution of the camera is 320 × 240 pixels in the MP4 format. Furthermore, in order to learn the motion patterns of the leaking drops and evaluate the proposed method, the video data are divided into training and test videos. A thermal image with colors of the demonstrator plant without leakage taken by IR camera is shown in Figure 1a. This figure shows the setting of the demonstrator plant. This image is a raw image with RAVI format without any compression effect resulted from MP4 format. The provided video data set is in MP4 format and a sample of a frame in MP4 with leakage in gray scale is presented in Figure 1b. The reason that the Figure 2b smeared is the effect of the background noise and the additional noise resulted from the compression in MP4 format.

In this study, the subsequent frames are subtracted to eliminate the effect of background and keep the effect of leaking drops (see Figure 1c). For better visualization of small leaking drops, pixels with zero values are shown as white and the pixels with high intensity are shown as black. As seen in Figure 1c, there is noise spreading over the subtracted frame, and the most intensive noise is along the pipes. The noise along the pipes can highly affect the analysis and avoid detecting the leaking drops correctly. However, the noise has a chaotic pattern in subsequent frames while the leaking drops follow a specific motion pattern. Therefore, in order to differentiate the noise from actual leaking drops in the images, the pattern for leaking drops should be identified. Before identifying the pattern of the leaking drops, the level of noise in subtracted frames can be reduced by applying background noise removal [29]. For this purpose, a threshold, $T_{a}$, can be considered and the pixels, whose absolute values are lower than this threshold, are set to zero (see Figure 1d). The remaining noise in the frames will be differentiated from the tracking objects by means of Kalman filter.

### 4.2. Typical Kalman Filter

Detection of leaking drops from different positions of the plant in a video can be modeled as the problem of tracking multiple objects in machine vision. This requires predicting the position of the tracking object in the following frames and matching it with the actual position of the object in these frames. In most of the cases, in a process plant, leaking drops from the pipelines move vertically downwards due to gravity. Therefore, they can be observed in a vertical line. This assumption cannot be held if the leakage is at the bottom part of the plant on the ground, in which the leakage appears as spreading liquid on the surface. In this paper, the focus is on the cases that leaking drops can be observed as moving objects in the vertical direction. Furthermore, the leakage in a video can be observed as a repetitive phenomenon. It starts from the starting point, it moves downwards through the several subsequent frames and it repeats again. Therefore, the whole video can be considered as several intervals (subsequent frames) representing a repetitive phenomenon. However, due to a variety of random noise caused by external forces (e.g., air friction) and the errors in cameras and video compression, the motion is not completely the same at different intervals. By considering these properties of leaking drops in a video, the motion of the leaking drops can be modeled as a linear stochastic system. In this system, the noise in the motion itself caused by external factors, and the noise in the measurements caused by measurement devices can be represented as process noise and measurement noise respectively, in the Kalman filter model [43]. For a linear motion, it has been proved that the Kalman filter is the stable and optimum model [43,44] and it has been used in tracking linear motions in different applications domain such as human motion tracking [45] and vehicle tracking [46] in recent years. Therefore, if the motion of the leaking drop can be modeled by a linear model, then the Kalman filter can provide an optimum estimation of the motion pattern (position, velocity, and acceleration) for the leaking drop and a stable prediction of its next position.

In order to estimate the internal states of this linear stochastic system, such as the position, velocity, and acceleration of a leaking drop, the discrete Kalman filter is utilized. It is not only computationally efficient and robust [36], but also theoretically precise, since the optimal state can be found with the smallest possible variance error, recursively. In comparison with other existing methods for object tracking in a video, such as a particle filter, the Kalman filter has better performance when the motion of the tracking object can be estimated as a linear process, and it is more robust to noise [34]. In Kalman filter, a discrete-time process can be described by a linear stochastic differential equation as:

(1) $$y_{k}=Ay_{k-1}+w_{k-1}$$

where $y_{k}$ represents the state at time stamp k, A is the state transition matrix that projects the state at time stamp k−1 to the state at time stamp k, and $w_{k-1}$ is the Gaussian process noise with normal distribution p(w)~N(0,Q). The actual measurement is assumed to occur at discrete time stamps and satisfies the linear relationship with the state:

(2) $$z_{k}=Hy_{k}+v_{k}$$

where $z_{k}$ is the actual measurement at time step k, H is the connection matrix and gives the ideal connection between the measurement $z_{k}$ and the state vector $y_{k}$, and $v_{k}$ is the Gaussian measurement noise with normal distribution p(v)~N(0,R). The discrete Kalman filter algorithm consists of two steps: prediction and correction. In the prediction step, the algorithm obtains the prior estimation of $y_{k}$, which is denoted as ${\hat{y}}_{k}^{-}$, based on the state transition matrix as follows:

(3) $${\hat{y}}_{k}^{-}=A{\hat{y}}_{k-1}+w_{k-1}$$

It is assumed that an initial estimation of the process at time stamp k−1 is known. After prediction, by substituting Equation (3) into the error covariance matrix, $P_{k}^{-}=E\left[\left(y_{k}-{\hat{y}}_{k}^{-}\right){\left(y_{k}-{\hat{y}}_{k}^{-}\right)}^{T}\right]$, the prediction error can be rewritten as $P_{k}^{-}=AP_{k-1}A^{T}+Q$.

In the correction step, the algorithm updates the estimation by incorporating the measurement into the prior estimation to obtain and update estimation, $\;{\hat{y}}_{k}$, as follows:

(4) $${\hat{y}}_{k}={\hat{y}}_{k}^{-}+K_{k}\left(z_{k}-H{\hat{y}}_{k}^{-}\right)$$

where $K_{k}$ is known as Kalman gain. By substituting Equation (4) into $P_{k\;}^{-}\;$and minimizing it, the Kalman gain, $K_{k}$, and updated error covariance matrix, $P_{k}$, can be calculated as:

(5) $$K_{k}=P_{k}^{-}H^{T}{\left(HP_{k}^{-}H^{T}+R\right)}^{-1}$$

(6) $$P_{k}=\left(1-K_{k}H\right)P_{k}^{-}$$

In the next subsection, the steps towards obtaining the Kalman filter model of the leaking drops are explained.

### 4.3. Calculation of Measurement Points in Training Set: Segmentation, Object Definition, and Feature Extraction

In this study, the training data are used to obtain the state of the Kalman filter. Since there are different leakages with different velocities in training videos, different Kalman filter models can be derived to capture all possible motion patterns of leakages in the training data. Then, the velocity and acceleration estimated from training videos can be used to predict the positions of leakages in a test video.

Particularly, leakages in this study move approximately vertically, whereby the x-coordinate (horizontal position of a leakage) remains constant during the entire movement. Therefore, only the motion along the *y*-axis is considered for analysis and is used as an actual measurement in the Kalman filter algorithm. To estimate the velocity and acceleration of leakages in training videos, it is essential to obtain the measurement as precisely as possible. In order to measure the y-coordinate of a leakage in a training video, suitable image segmentation is required [8]. For this purpose, the subtracted frames are divided into vertical strips with size 240× α pixels, where *α* is the width of each strip. Dividing the frames to the strips limits the search area for tracking the leaking drops as objects. This division will reduce the time complexity of the algorithm, and instead of searching for the next possible position of the tracked object (leaking drop) in the entire frame, a limited area will be searched.

The strips including the leaking drops in a training video are processed individually to obtain the Kalman models. This means that if in a video there are n leakage positions, n different Kalman filters should be defined for n different leakage patterns. In other words, each leakage is considered as a single object tracking problem, and therefore multiple leakages are considered as multiple object tracking. In general, if there are L different videos in training data including leakages, there are *N_P_* = *L* × *n* different leakage patterns in the training data, and therefore $N_{P}$ Kalman filters are required. Furthermore, since the leakage is a repetitive phenomenon, each training video is divided into the intervals (including f frames), in which each interval shows a complete motion of a single drop from the top to the bottom of the frame. This interval is referred as the tracking window to track one single drop from a specific leakage. The value of f in each tracking window (the length of tracking window) is different per each individual leakage in the training videos and it depends on the pattern (positions and velocity) of the individual leakage. This number can be obtained by observing training videos per each individual leakage and checking how many frames are required until a single drop reaches the bottom of the frame. Therefore, different tracking windows are considered to track each individual leakage in training videos.

In order to track leaking drops in an individual leakage during a tracking window, it is necessary to define a single leaking drop as an object. Since the leaking drops do not have specific shape or size, it is challenging to define them as objects in the noisy frames. There are several techniques to define a moving object in the existing literature [30]. One possible technique to define the leaking drops as objects is to consider a probability density appearance model for leaking drops [30]. In this model, a probability density function such as the Gaussian model is defined to model the pixels which form an object. However, this object definition is not suitable for leaking drops. One reason is that in thermographic images, when the leaking drop reaches the bottom of the frame, it loses the temperature, and therefore the intensity of the pixels which form a leaking drop is changed while the object is moving in subsequent frames. This is referred to as the fading problem. Therefore, one single drop does not have the same probability density appearance model in subsequent frames (see Figure 2a). Another possible and pragmatic way to define the leaking drops as objects is to define them as a set of neighboring points [30]. In this case, it is assumed that a leaking drop is a non-zero pixel with at least h and g neighbors in the vertical and horizontal directions, respectively (see Figure 2b)

**Figure 3:**
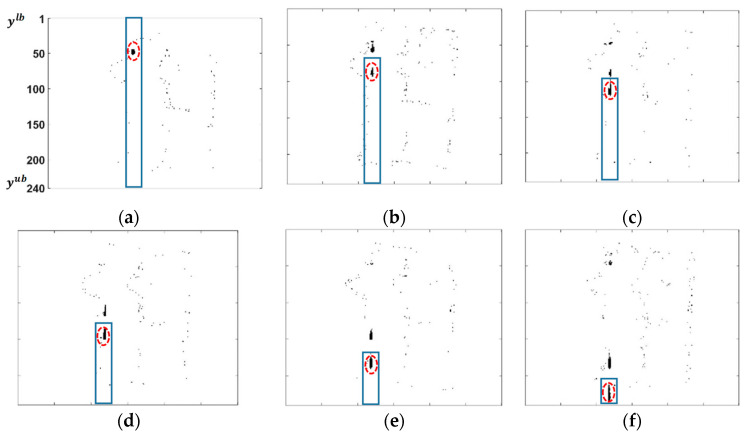
(**a**–**f**) Subsequent subtracted frames during an interval. The leaking drop is tracked based on its intesity and its neighbors along the strip. The lower boundry of the strip is increased to correctly measure the next position of the leaking drop.The tracked leaking drop is marked with dash-line circle.

The steps for tracking a leaking drop are shown in Figure 3. Assume that the upper bound and the lower bound of the strip in y-coordinate is known as $\left[y^{lb},y^{ub}\right]$, which is (1, 240) at the beginning (the index of the pixels in the vertical direction is increasing from the top of the frame to the bottom, see Figure 3a). In order to measure the first position of the leakage in the first frame of the tracking window, the pixel with the highest intensity, which has at least h and g neighbors in the vertical and horizontal directions respectively, is selected. Furthermore, as an additional feature to form and differentiate the leaking drop as an object from the noise, the relative mass of the detected pixel and its neighbors to the mass of the whole strip is considered as well. Assume that C is the matrix, which shows the intensity of pixels in the strip. If in this matrix *c_i,j_* shows the intensity of a pixel in position i and j, then the average mass in the strip, $m_{c}$, can be calculated as follows:

(7) $$m_{c}=\frac{\sum_{i=y^{lb}}^{y^{ub}}\sum_{j=1}^{\alpha }c_{i,j}\;}{\alpha \cdot \left(y^{ub}-y^{lb}\right)}$$

Assume that the average mass of the most intensive pixel with its neighbors is$\;m_{s}$. If$\;m_{s}\geq \xi \cdot m_{c}$, 0.5<ξ<1, then the selected point can be considered as a leaking drop. This additional feature means that the pixels which form a leaking drop as an object should have a reasonable intensity as well, which can be compared relatively to the average intensity of pixels in the entire strip. In order to measure the position of the leaking drop, the center of mass of the detected leaking drop as an object is considered in the measurements as follows:

(8) $$z_{1}=y^{lb}+\frac{\sum_{i=1}^{h}\sum_{j=1}^{g}i\cdot s_{i,j}\;}{\sum_{i=1}^{h}\sum_{j=1}^{g}s_{i,j}\;}$$

where $s_{i,j}$ is the intensity of each pixel in the vicinity of the selected pixel. In order to compute the second measurement point of the leakage, a similar process is repeated in the next frame in the tracking window, but with a change in the dimension of the strip (see Figure 3b). In the next frame, the next candidate pixel to track the same leaking drop is searched below the first measured position of the leaking drop. Therefore, the lower bound of the strip is increased to $y_{new}^{lb}=\;y^{lb}+z_{1}+\;\delta$, where δ is selected as a small number of pixels to make sure the new lower bound is lower than the first measured position. This process is repeated until the last frame in the tracking window. In order to get the new measurements for the same single drop, the next tracking window for the same leakage is processed as well. An example of different measurements (position of tracking object) in four tracking windows for a single drop in one selected video are listed in Table 2.

In this table, each row represents the measurement positions of a leaking drop in a tracking window. As seen, the leakage in this strip appears repeatedly and its motion lasts for approximately 6 frames. However, note that the fourth row has significantly fewer measurements (the leaking drop is not observed at the two last positions) and the values deviate highly from the other values in the same column. Noisy pixels are likely mistaken as an object or the intensity of the leakage at frames four and five is too low to be observed due to fading. To obtain an accurate mean value of the measurements, outliers such as $y_{4}$ in the fourth row should be removed in advance. The outliers are detected using the interquartile range (IQR). Outliers are defined as values that fall outside of either 1.5×IQR below the first quartile or 1.5×IQR above the third quartile. In this case, IQR is applied for each column in Table 1. Afterwards, the mean value of each column is computed and used as the measurement input of the Kalman filter, and the variance in each column ($y_{k}$) is considered as the measurement error, $v_{k}$, in this model.

### 4.4. Estimation of Velocity and Acceleration for Each Leakage Pattern in Training Set

The obtained measurements can be used to estimate velocity, $\dot{y}$, and acceleration, $\ddot{y}$, by applying the Kalman filter for each leakage pattern individually. Based on linear motion Equations (1) and (2) can be rewritten as follows:

(9) $${\left[\begin{array}{c} y\\ \dot{y}\\ \ddot{y} \end{array}\right]}_{k}=\underset{A}{\underbrace{\left[\begin{array}{ccc} 1 & \Delta t & 0.5\Delta t^{2}\\ 0 & 1 & \Delta t\\ 0 & 0 & 1 \end{array}\right]}}{\left[\begin{array}{c} y\\ \dot{y}\\ \ddot{y} \end{array}\right]}_{k-1}+\;{\left[\begin{array}{c} 0.5\Delta t^{2}\\ \Delta t\\ 1 \end{array}\right]}_{k-1}\sigma^{2}$$

(10) $$z_{k}=\underset{H}{\underbrace{\left[\begin{array}{ccc} 1 & 0 & 0 \end{array}\right]}}{\left[\begin{array}{c} y\\ \dot{y}\\ \ddot{y} \end{array}\right]}_{k-1}+v_{k}$$

where Δt is the time difference between two consequent observation of $y_{k-1}$ and $y_{k},\;$and defined as Δt=1. $\sigma^{2}$ is the variance of the process error and it is assumed as a constant. Since the measurements are only available for positions, H is considered as $\left[\begin{array}{ccc} 1 & 0 & 0 \end{array}\right]$ and measurement error,$\;v_{k}$, is calculated by means of variance in different measurements of $y_{k}$ in different tracking windows. Based on the state Equation (9), measurement Equation (10), and Kalman Equations (4)–(6), the velocity and acceleration of the motion can be estimated as well. Finally, the estimated model for the motion can be considered as the motion pattern of a leaking drop as follows:

(11) $$y_{k}=y_{k-1}+{\dot{y}}_{k-1}\Delta t+\frac{1}{2}{\ddot{y}}_{k-1}\Delta t^{2}$$

**Figure 4:**
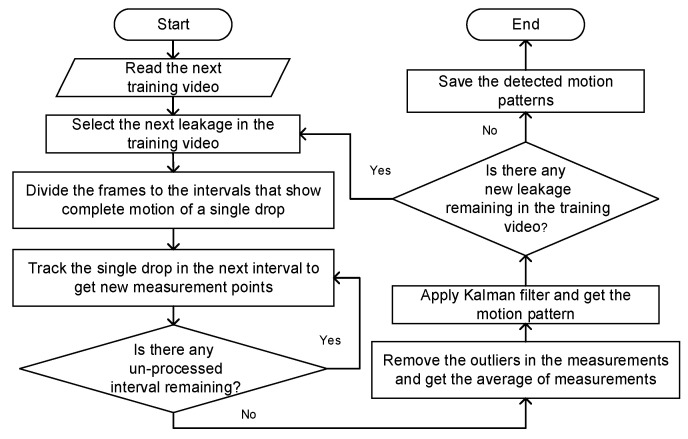
Steps to obtain the measurement points of leakage positions for Kalman filter and motion pattern detection in training videos.

This model can be derived for any leakage in the training videos, and therefore $N_{P}$ individual models the same as Equation (11) can be derived for all possible leakage patterns in the training videos. A summary of the steps of the proposed algorithm for motion pattern detection of leaking drops is presented in Figure 4.

### 4.5. Leakage Detection in Test Video: Possible Positions and Predicted Positions

The obtained patterns from the training videos can be used to check for leakages in test videos. First of all, the test video is divided into the frames and subtracted frames are used for the analysis. Since the positions of the leaking drops are not known in a test video, all the strips with the width α should be searched to detect the possible leakage area. In each strip, the *possible positions* for the leakage are detected by a similar method introduced for the calculation of measurement points in the training video in subsection *C*. Since the length of the tracking windows is not known in test data, the possible positions for a leaking drop are searched frame by frame until the bottom of the strip. When the search reaches the bottom of the strip, one set of possible positions for a leaking drop is determined. Since leakage is a repeating process and can be observed again in the next frames, the second set of possible positions for the same leaking drop can be determined again. In order to obtain a set of possible positions of the leakage, the test video is parsed for γ subsequent frames. It is assumed that these γ frames include w tracking windows. Each tracking window includes several positions of the leaking drop in some subsequent frames. Assume that the first tracking window includes *f*_1_ frames. In this case, $Y_{1}^{possible}=\left[\begin{array}{cccc} y_{1}^{possible} & y_{2}^{possible} & \cdots & y_{f_{1}}^{possible} \end{array}\right],$ shows the possible positions of the leaking drop in the first tracking window. When the position tracking reaches the end of the frame, a new tracking window is started. After γ frames, several measurement vectors (resulted from several tracking windows) are available for the possible positions of a leaking drop as follows:

(12) $$\begin{array}{c} Y_{1}^{possible}=\left[\begin{array}{cccc} y_{1}^{possible} & y_{2}^{possible} & \cdots & y_{f_{1}}^{possible} \end{array}\right],\\ Y_{2}^{possible}=\left[\begin{array}{cccc} y_{1}^{possible} & y_{2}^{possible} & \cdots & y_{f_{2}}^{possible} \end{array}\right],\\ Y_{w}^{possible}=\left[\begin{array}{cccc} y_{1}^{possible} & y_{2}^{possible} & \cdots & y_{f_{w}}^{possible} \end{array}\right]. \end{array}$$

where w is the number of tracking windows within γ frames. The tracking windows [1,…,w] include $f_{1}$, $f_{2}$,…,$\;f_{w}$ frames, respectively. In order to take the average over all detected possible positions, $f=\max\left(f_{1},\;f_{2},\ldots ,\;f_{w}\right)$ is selected, outliers in each column are removed, and the average of each column is calculated for existing values in the corresponding column. The final vector of possible positions in the selected strip is referred as $Y^{possible}=\left[\begin{array}{cccc} y_{1}^{possible} & y_{2}^{possible} & \cdots & y_{f}^{possible} \end{array}\right]$. After determining the possible positions, the *predicted positions* are calculated by the obtained Kalman filter models from training videos using Equation (11).

In order to assign the detected possible positions to the predicted positions, the following process is proposed. Assume that the predicted position by one of the obtained Kalman filters is as follows:

(13) $$Y^{predict}=\left[\begin{array}{cccc} y_{1}^{predict} & y_{2}^{predict} & \cdots & y_{p}^{predict} \end{array}\right]$$

For a measurement $y_{l}^{possible}$ and a predicted $y_{m}^{predict}$, if they satisfy:

(14) $$\left|y_{m}^{predict}-y_{l}^{possible}\right|\leq T_{match},l=1,\ldots ,f,\;m=1,\ldots ,p$$

they are denoted as a matching pair of positions and excluded from the rest of comparison. In this matching, $T_{match}$ is a threshold to compensate for errors in measurements in the test video. After the comparison between all the pairs, the number of matching pairs is counted and denoted as $n^{match}$. The matching ratio $r^{match}$ for this group of measurements and predictions is computed as $r^{match}=n^{match}/p$. This ratio is computed for all obtained Kalman filters as well. If the highest matching ratio after comparison with all obtained Kalman filters, $r_{max}^{match}$, is more than 80%, the possible positions in the corresponding strip in the test video are marked as leakage, otherwise the detected possible positions are considered as noise and will be ignored. This process is done for all strips in the test video. The proposed process is relatively robust to the occurring noise. In fact, the defined matching ratio ($r^{match}$) avoids assigning noisy pixels to the predicted positions. This ratio ensures that more than 80% of the detected possible positions are compatible with the predicted positions by a Kalman filter in the tracking windows along each vertical stipe. In the proposed method in this paper, dividing the image to the vertical stripes and searching for the next possible position of the leakage in the same stripe reduces the search space. Furthermore, the matching ratio ($r^{match}$) in the same stripe ensures robustness to noise while other methods such as the Hungarian association algorithm are more sensitive to the noise [42].

### 4.6. Leakage Detection in Test Video: Particle Filter as a Baseline Method

One of the most challenging problems in leakage detection from the pipelines is to differentiate the actual small leaking drops from the environmental noise. In this case, a promising approach is to show that the leaking drops have a certain motion pattern while the noise does not follow any specific pattern. The introduced Multi Kalman filter approach tries to learn the motion pattern from training data and tune the parameters of the motion such as velocity and accelerator. These patterns are applied to the test data to differentiate the leakage from the noise.

In order to evaluate the performance of the proposed method, a particle filter as a non-parametric method is applied to the leakage detection as well. Since the particle filter is an online and non-parametric method and does not require training to learn specific parameters, it is considered a baseline approach for evaluation of the performance of the proposed method. The comparison between proposed method and the particle filter as a general model for motion tracking can provide an overview of pros and cons of the proposed Multi Kalman filter model.

In the case of leakage detection, the particle filter is directly applied to the test data. For this purpose, the possible positions measured in test data, $Y^{possible}=\left[\begin{array}{cccc} y_{1}^{possible} & y_{2}^{possible} & \cdots & y_{f}^{possible} \end{array}\right]$, introduced in a previous section, are used. Since there is no pattern detection step in a particle filter, there are not any predicted positions available from the training model in the case of a particle filter. Therefore, a comparison between the possible positions and the predicted positions is not possible. According to the particle filter algorithm [34], the method generates a set of samples which are called particles and uses these particles to estimate the state of the system. The state of each particle is updated in each iteration of the algorithm. At the end of each iteration, the particles which can estimate the target measurement positions more precisely get higher weights than the other particles, and the particles with higher weights are selected for resampling for the next iteration. In the case of leakage detection, $Y^{possible}$ is considered as the target measurement points in which the generated particles in the particle filter model should estimate these measurement points.

Suppose that $M_{pf}$ is the number of particles in a particle filter model and $x_{t}^{m_{pf}}$ is the state (position) of the particle $m_{pf},\;1\leq m_{pf}\leq M_{pf}$, at iteration t of the particle filter algorithm. For simplicity, this position is considered as the vertical position of particles in the frames, since the leaking drops move vertically. In the particle filter model, there are two main steps in each iteration of the algorithm: the *prediction* step and the *correction* step. In the prediction step, $x_{t}^{m_{pf}}\;$is sampled from the probability $P(x_{t}^{m_{pf}}|x_{t-1}^{m_{pf}},\;u_{t}^{m_{pf}})$, where $x_{t-1}^{m_{pf}}$ is state of the particle at the iteration t−1 and $u_{t}^{m_{pf}}$ is the control parameter. In this paper, it is assumed that $P\left(x_{t}^{m_{pf}}|x_{t-1}^{m_{pf}},\;u_{t}^{m_{pf}}\right)=N\left(x_{t-1}^{m_{pf}},u_{t}^{m_{pf}}\right)$, which is the normal distribution centered in $x_{t-1}^{m_{pf}}$ with variance $u_{t}^{m_{pf}}.$

In the correction step, the distance between the measurement points, $Y^{possible}$, and the current state of each particle $x_{t}^{m_{pf}}$ is calculated. According to this distance, each particle gets an importance weight. This importance weight is calculated as $w_{t}^{m_{pf}}=P\left(Y^{possible}|x_{t}^{m_{pf}}\right)$. This weight assignment means that the estimated state $x_{t}^{m_{pf}}$, which is closer to the measurement positions $Y^{possible}$ gets a higher weight. In the next iteration, the particles are resampled based on their weights and the particles with higher importance weight are selected for the next state estimation step. For this purpose, each importance weight $w_{t}^{m_{pf}}$ should be normalized according to ${\tilde{w}}_{t}^{m_{pf}}=\frac{w_{t}^{m_{pf}}}{\sum_{i=1}^{M_{pf}}w_{t}^{i}}$. In fact, the particle $x_{t}^{m_{pf}}$ is selected with probability ${\tilde{w}}_{t}^{m_{pf}}$ for the next iteration. Finally, the selected particles with high weights are fit to the measurement positions, and therefore they can track the leakage.

In the evaluation section, the proposed multi Kalman filter method and the particle filter both are applied to the leakage detection problem and are compared regarding their performance.

## 5. Evaluation of the Proposed Method in Leakage Detection and Localization

In this section, the accuracy of the proposed method for leakage detection in test videos is evaluated first. Then, the possibilities and limitations for extending the method for real industrial applications are discussed.

### Evaluation of the Accuracy of the Proposed Method for Leakage Detection in Test Videos

In order to evaluate the proposed method, a testbed as described in Section 4.1 is set up (see Figure 1a). In this testbed environment, an IR camera is used to capture video data of the complete demonstrator plant and this data is used for image analysis. The resolution of the camera is 320 × 240 and it has temperature resolution of 75 mK. The format of the video data is MP4, it is compressed video, and data were captured for one minute during each measurement. During all measurements, the pumped liquid inside the pipelines is water with a regulated temperature of about 40–45 °C, while the testbed is operated at room temperature. At first, some video footage was taken while the plant was in normal operation (without leakage). These videos are called “normal videos”. Then, to provide a dataset for model training, different liquid leakages with different leakage speeds and positions in the demonstrator plant are provided. These videos are called “anomalous videos”. In these videos, the leakage is generated by opening the provided small valves in the demonstrator or by losing distinct pipe connections in different positions.

The data obtained from the IR camera are divided into training and test videos. The training data are used for model training and test data are used for evaluation of the model. The data set includes 23 videos, eight normal videos, and fifteen anomalous videos with different leakages. For training, nine anomalous videos are used since there are no objects to track in normal videos. For the test data, four normal videos and six anomalous videos are used. The leakage tracking is done by the proposed Kalman filter model as well as the particle filter as the baseline model. In order to evaluate the effectiveness of the proposed method for leakage detection, the classification results are investigated in terms of accuracy, misclassification, F_1 score, recall, and precision for test data. In this evaluation, the values for defined parameters for the proposed method and also for the particle filter are summarized in Table 3.

In the parameter setting in Table 2, $T_{a}$ is the threshold for background noise. Increasing the value of $T_{a}$, will affect the leakage pattern detection, especially at the bottom of the frames when the leaking drops lose the temperature and have less intensity. α is the width of strips in the frames. By increasing the value of α, one strip might include more than one leakage which will affect the detection of them, and decreasing the α, will increase the time complexity by searching very small strips that do not include any leakages. The selected value for α can be adjusted to the approximate size of the leaking drop. In this paper, the minimum size of a leaking drop is considered as h×g=5×3 (minimum size of the tracked object), which is the minimum number of neighboring pixels in vertical and horizontal directions of the pixel with the highest intensity in the strip in the object definition process. Therefore, the selection of α=10 can help to find larger leaking drops (approximately three times larger than the minimum size) in the strip as well. ξ is selected as 0.7, which means the average mass of the detected object (leaking drop) should be at least 70% of the average mass in the strip; otherwise, the leaking drop is not properly observable. The variance of process error is considered as $\sigma^{2}=9$, which means it is assumed that the new measurement position of the object, $y_{k}$, can be affected by process noise of approximately 4 or 5 pixels based on Equation (9). The number of frames for searching leakages in test data is considered as γ=50, and if no leakage is detected in the first 50 frames, the search will continue for the next 50 frames. Finally, the $T_{match}$ for matching (assigning) the possible positions of leakage in test data with predicted positions by Kalman filter model is considered as $T_{match}=10$. Furthermore, for the particle filter model, the initial number of particles are selected as $M_{pf}=20,000\;$and $u_{t}^{m_{pf}}=2$. For implementing the particle filter, the Robotic System Toolbox of Matlab 2020a (MathWorks, Munich, Germany) is used.

**Figure 5:**
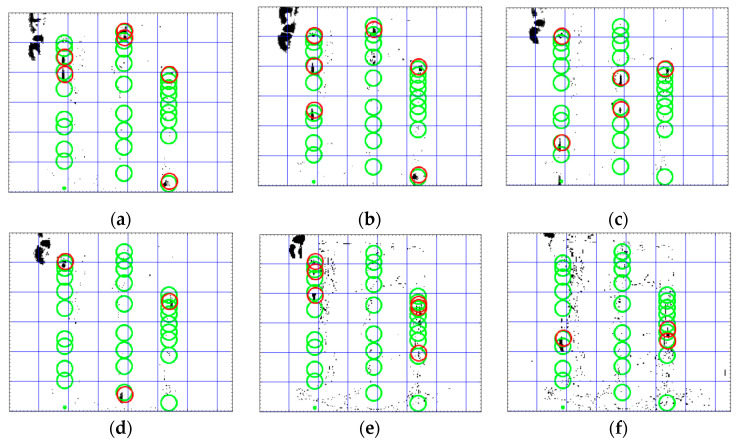
Detected leakage in test videos using the proposed multi Kalman filter method in six subsequent frames (**a**–**f**) in Video 6. The green circles show the matched points between positions predicted by Kalman filters and the measured possible positions in test data. The red circles show the current position of the leakage in the each frame.

**Figure 6:**
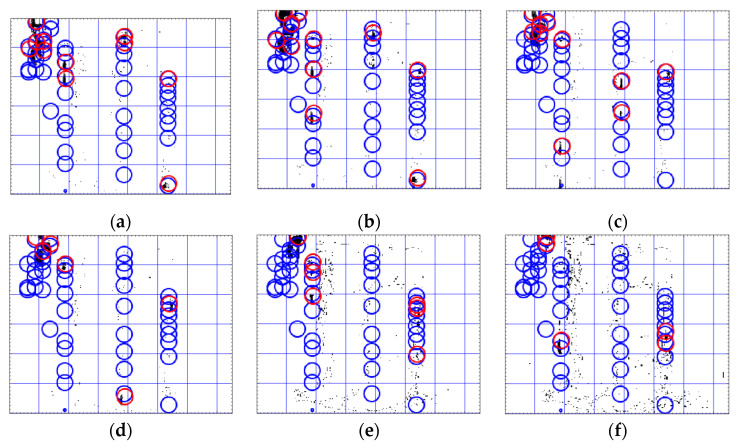
Detected leakage in test videos using the proposed multi Kalman filter method in six subsequent frames (**a**–**f**) in Video 6. The green circles show the matched points between positions predicted by Kalman filters and the measured possible positions in test data. The red circles show the current position of the leakage in the each frame.

To perform the evaluation, each frame in a test video is divided into blocks with a size of 40×40 pixels (see Figure 5 and Figure 6). For the classification purpose, the actual class of each block is marked as normal (positive) if there is not any leaking drop in that area, and is marked as anomalous (negative) if the block includes leakage (or is expected to include leakage due to the path of the leaking drops). The results of the classification of normal (positive) videos and anomalous videos for the test data and the accuracy of the classification are summarized in Table 4 and Table 5 for the proposed multi Kalman filter method and the particle filter as baseline method, respectively. Since the localization of the leakage is important in this paper, each video is divided into regions (blocks) and the classification is applied on each video region-wise. This classification makes sure that in one single video, all the regions including leakage can be detected. If the normal region is detected as normal, then it is considered as True Positive (TP). If the normal region is detected as the leakage region, then it is considered as False Negative (FN). If the leakage region is detected as the normal region, then it is considered as False Positive (FP). Finally, if the leakage region is detected as the normal region, it is considered as True Negative (TN). These numbers are given in Table 4 and Table 5. The numbers in these tables show how many regions in one single video are classified correctly.

In all normal videos, the Kalman filter method classifies the intensive pixels as noise correctly. The noise arises at random and is not repeated at the positions regularly. Therefore, the measurements of possible positions deviate from the predicted positions significantly. The deviation of the measurement positions and the predicted positions leads to lower matching ratio. In this case, the measurement positions are considered as noise by the algorithm and not assigned to the leakage. However, in particle filter, since there is not any learnt pattern in advance for prediction and comparison, the noisy normal videos are classified as anomalous videos and the noisy blocks are marked as the leakage in these normal videos (see Videos 3 and 4 in Table 4).

**Figure 7:**
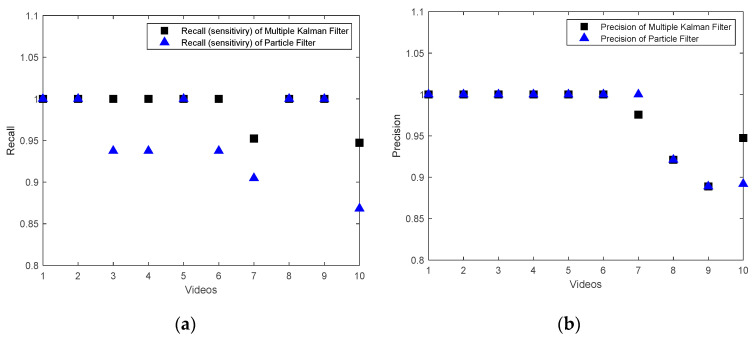
Recall (**a**) and Precision (**b**) of the proposed multi Kalman filter method and the particle filter for the test videos.

**Figure 8:**
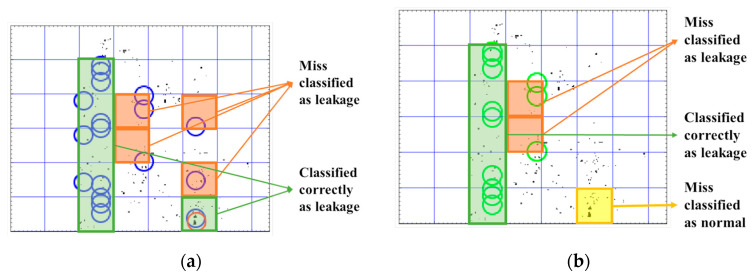
One sample frame in Video 7. (**a**) Particle filter can detect the one small leakage at the bottom of the frame. However, it has more misclassifications due to tracking the noise as well. (**b**) Proposed multi Kalman filter method has less misclassification and is more robust to the noise. However, it is missing one single drop at the bottom of the frame.

In the anomalous videos, the leakage can be detected with an accuracy of more than 90% in all areas that include leakages in both the proposed Kalman filter method and the particle filter (see Videos 5–10 in Table 4 and Table 5). However, the particle filter method detects the noise in anomalous videos as leakage more than the Kalman filter. This situation for several subsequent frames for Video 6 are shown in Figure 5 and Figure 6. In the proposed Kalman filter model (Figure 5), the possible positions which are matched with Kalman filter models are marked as green circles. These green circles show the trajectory of the leakage. The current position of the leakage in each frame is marked as red. In the particle filter model (Figure 6), the final positions detected by the particle filter as the positions of leakages are marked as blue circles and the red circles are the current position of the object in the current frame. As seen in Figure 6, the particle filter algorithm tracks the noise as well as the leakages. Therefore, the particle filter is more sensitive to noise and generates more false alarm than the proposed multi Kalman filter method. In order to compare the sensitive to noise, the recall (sensitivity) metric, $Recall=\frac{TP}{TP+FP}$, and precision metric, $Precision=\;\frac{TP}{TP+FP}$, of both methods are compared in Figure 7. The recall metric in Figure 7a shows the sensitivity of particle filter to the noise. Even though this method can detect the leakages, it generates more false alarms than the proposed method in this paper. The precision metric in Figure 7b shows that in one video (Video 7), the proposed Kalman filter classifies one leakage block as normal (see Table 3). The reason for this misclassification is that the leakage position is at the bottom of the frame, and therefore the Kalman filter cannot track it since there is enough measurements along the frame. The misclassification in Video 7 in one sample frame is shown in Figure 8.

In summary, all videos are classified correctly by using the proposed multi Kalman filter method, although small areas of the trajectory are overlooked by the algorithm. Therefore, if the motion of the leakage can be observed in the video, the method can detect it with high accuracy (R1). According to the Table 4, in videos with leakages (anomalous videos), the regions (blocks) including leakages and regions without leakages (normal) are detected with an accuracy of more than 90%. Furthermore, all the regions in the normal videos without leakage are classified as normal as well with 100% accuracy. These results show that the proposed method can differentiate the noise from actual motion (R2) as well. The fulfillment of Requirement R2 is also shown in Figure 5 (in comparison with Figure 6). However, in Video 7 and Video 10 in Table 4, a few normal blocks are classified as anomalous blocks which can indicate the FN classification of these blocks. This shows that if the videos are highly noisy, it will affect the accuracy of the proposed method and lead to some misclassification and high FN rate.

According to Figure 5, the proposed method can also detect the small drops in a noisy environment (R3). However, from Videos 7–10 in Table 4, there are a few anomalous regions (blocks) with leakage which are classified as normal blocks which can indicate the FP classification of these blocks. The main reason for such misclassification is that the leakage location is at the bottom of the frames, and therefore it is not possible to track the trajectory of the leakage. Therefore, it is not possible to get enough measurement positions to apply the proposed method and track the motion. This situation is shown in Figure 8b. Even though some regions with leakage are classified as normal region, still in these videos (Videos 7–10) the majority of the blocks including the leakages (anomalous) are classified correctly as leakage (anomalous) which shows the high TN rate. The TN rates ($\frac{TN}{number\;of\;negative}$) for Videos 7–10 are 71.5%, 77%, 75%, and 80%, respectively. The size of detected drops is 3×5 pixels, which is relatively small in comparison with the size of the whole frame (320×240). Due to dividing the image into strips and having multiple Kalman models, several simultaneous leakages in the video can be detected (R4). By the method proposed for possible position detection and matching them with predicted positions, the leakage is localized and its trajectory is tracked as well (R5). The only case that the trajectory cannot be tracked is when the leakage position is at the bottom of the frame. The prior leakage detection method proposed by the authors on the same dataset [29] can only detect the videos including leakage. In comparison with [29], the proposed method in this paper not only can detect the videos including leakages but also can localize and track the leakages in each video. Finally, since the physical properties of the liquid and pipes are not considered here, it can be applicable for different types of liquid and material of the pipelines (R6).

## 6. Conclusions and Outlook

In this paper, an approach based on motion pattern detection using the Kalman filter is proposed to detect leakages from pipelines in a process plant. In order to apply leakage detection, a testbed including a demonstrator plant and an IR camera is used. The resolution of the camera is 320 × 240 pixels and the minimum size of the leaking drop in such an image was 5 × 3 pixels. The camera is placed in front of the plant, and in each video different leakages from different positions of the plant are generated. The proposed method could capture the motion patterns of leaking drops in training videos and can be further used for leakage detection and localization in test videos. The results show that the proposed method can detect leakages in test videos with accuracy higher than 90%. Furthermore, a particle filter is used as a baseline model to compare and evaluate the performance of the proposed method in terms of accuracy and misclassification. The results show that the proposed method has less sensitivity to noise and can differentiate the leakage from noise more precisely than a particle filter. Further research on this topic will focus on extending the method to apply for a real large-scale process plant. One way to apply the proposed method to real large-scale plants is the projection of the observed position in the screen to the real position of the leaking drops. For this purpose, the angle of the camera and the distance of the camera to the plant should be included in the analysis as well. This can be achieved by calibrating the camera, using geometry and projecting techniques and estimating the depth in images. Additionally, different patterns of leakage, such as spreading liquid on the surface as another type of leakage, can be included in the image analysis as well. In this case, methods such as region growing and Gaussian models for the probability density appearance model can be used to detect this type of leakage.

Furthermore, in real application in an indoor large-scale plant, considering several fixed cameras in front of the plant can be a way of implementing the proposed visual inspection method. Each camera can observe the specific part of the plant (as a single plant) and the vision-based algorithm is implemented for each specific part of the plant that is observed by a single camera. For instance, if the plant is in dimension of 10 m × 70 m, it is not possible to film the entire plant and detect the leakage. In such a case, the cameras should be installed close to the pipes and valves. However, if the plant is huge, the possibility of installing a lot of cameras to observe all parts of the plant should be investigated as well.

## Figures and Tables

**Table 1:** Summary of the requirements.

	R1	R2	R3	R4	R5	R6
Summary of the requirements	High accuracy	Robust to the noise	Detection of small drops	Multiple leakages	Leakage localization	Independent of physical properties

**Table 2:** An Example of Measurement Results of a Single Leaking Drop in Four Subsequent Tracking Windows in a Training Video. Shading is to differentiate the row.

Position of the Leakage in Vertical Direction						
	$y_{1}\;$	$y_{2}\;$	$y_{3}\;$	$y_{4}\;$	$y_{5}\;$	$y_{6}\;$
Tracking Window 1	47	85	115	148	185	225
Tracking Window 2	46	88	114	150	182	221
Tracking Window 3	46	82	118	147	189	224
Tracking Window 4	47	91	120	196	-	-

**Table 3:** Selected Values for the Defined Parameters.

Parameters	$T_{a}$	α	h×g	ξ	$\sigma^{2}$	γ	$T_{match}$	$M_{pf}$	$u_{t}^{m_{pf}}$
**value**	0.5	10	5 × 3	0.7	9	50	10	20,000	2

**Table 4:** Actual class, predicted class, and accuracy (acc. in percentage) of the classification for test videos, proposed multi Kalman filter. In this table, Normal represents a region (block) without leakage (positive) and Anomalous represents a region (block) with leakage (negative). Shading is to differentiate the rows.

				Actual Class			
Normal Videos				*Normal*	*Anomalous*	*Acc.*	*F1*
	Video 1	**Predicted class**	*Normal*	48 (100%)	0 (0%)	100	1
			*Anomalous*	0 (0%)	0 (0%)		
	Video 2	**Predicted class**	*Normal*	48 (100%)	0 (0%)	100	1
			*Anomalous*	0 (0%)	0 (0%)		
	Video 3	**Predicted class**	*Normal*	48 (100%)	0 (0%)	100	1
			*Anomalous*	0 (0%)	0 (0%)		
	Video 4	**Predicted class**	*Normal*	48 (100%)	0 (0%)	100	1
			*Anomalous*	0 (0%)	0 (0%)		
Anomalous Videos	Video 5	**Predicted class**	*Normal*	40 (83%)	0 (0.0%)	100	1
			*Anomalous*	0 (0.0%)	8 (17%)		
	Video 6	**Predicted class**	*Normal*	32 (67%)	0 (0.0%)	100	1
			*Anomalous*	0 (0.0%)	16 (33%)		
	Video 7	**Predicted class**	*Normal*	40 (83%)	1 (2%)	93	0.96
			*Anomalous*	2 (4%)	5 (11%)		
	Video 8	**Predicted class**	*Normal*	35 (72%)	0 (6%)	93	0.95
			*Anomalous*	3 (6%)	10 (20%)		
	Video 9	**Predicted class**	*Normal*	32 (67%)	0 (6%)	91	0.94
			*Anomalous*	4 (8%)	12 (25%)		
	Video 10	**Predicted class**	*Normal*	36 (75%)	2 (4%)	91	0.94
			*Anomalous*	2 (4%)	8 (17%)		

**Table 5:** Actual class, predicted class and accuracy (acc. in percentage) of the classification for test videos, proposed particle filter. In this table, Normal represents a region (block) without leakage (positive) and Anomalous represents a region (block) with leakage (negative). Shading is to differentiate the rows.

				Actual Class			
Normal Videos				*Normal*	*Anomalous*	*Acc.*	*F1*
	Video 1	**Predicted class**	*Normal*	48 (100%)	0 (0%)	100	1
			*Anomalous*	0 (0%)	0 (0%)		
	Video 2	**Predicted class**	*Normal*	48 (100%)	0 (0%)	100	1
			*Anomalous*	0 (0%)	0 (0%)		
	Video 3	**Predicted class**	*Normal*	48 (100%)	0 (0%)	93	0.96
			*Anomalous*	0 (0%)	0 (0%)		
	Video 4	**Predicted class**	*Normal*	48 (100%)	0 (0%)	93	0.96
			*Anomalous*	0 (0%)	0 (0%)		
Anomalous Videos	Video 5	**Predicted class**	*Normal*	40 (93%)	0 (0.0%)	100	1
			*Anomalous*	0 (0.0%)	8 (17%)		
	Video 6	**Predicted class**	*Normal*	30 (62%)	2 (4%)	95	0.96
			*Anomalous*	0 (0.0%)	16 (34%)		
	Video 7	**Predicted class**	*Normal*	38 (80%)	4 (8%)	91	0.95
			*Anomalous*	0 (0.0%)	6 (12%)		
	Video 8	**Predicted class**	*Normal*	35 (73%)	0 (0.0%)	93	0.95
			*Anomalous*	3 (6%)	10 (21%)		
	Video 9	**Predicted class**	*Normal*	32 (67%)	4 (8%)	91	0.94
			*Anomalous*	0 (0.0%)	12 (25%)		
	Video 10	**Predicted class**	*Normal*	33 (69%)	1 (2%)	87	0.91
			*Anomalous*	5 (10%)	9 (19%)		
